# The Influence of Signal Polarization on Quantum Bit Error Rate for Subcarrier Wave Quantum Key Distribution Protocol

**DOI:** 10.3390/e22121393

**Published:** 2020-12-09

**Authors:** Andrei Gaidash, Anton Kozubov, Svetlana Medvedeva, George Miroshnichenko

**Affiliations:** 1 Department of Mathematical Methods for Quantum Technologies, Steklov Mathematical Institute of Russian Academy of Sciences, Moscow 119991, Russia; andrei_gaidash@corp.ifmo.ru (A.G.); kozubov.anton@gmail.com (A.K.); 2 Laboratory of Quantum Processes and Measurements, ITMO University, Saint Petersburg 199034, Russia; 3 Faculty of Laser Photonics and Optoelectronics, ITMO University, Saint Petersburg 197101, Russia; gpmirosh@gmail.com

**Keywords:** quantum optics, quantum key distribution, polarization, Stokes parameters, dissipative dynamics

## Abstract

In this paper, we consider the influence of a divergence of polarization of a quantum signal transmitted through an optical fiber channel on the quantum bit error rate of the subcarrier wave quantum key distribution protocol. Firstly, we investigate the dependence of the optical power of the signal on the modulation indices’ difference after the second phase modulation of the signal. Then we consider the Liouville equation with regard to relaxation in order to develop expressions of the dynamics of the Stokes parameters. As a result, we propose a model that describes quantum bit error rate for the subcarrier wave quantum key distribution depending on the characteristics of the optical fiber. Finally, we propose several methods for minimizing quantum bit error rate.

## 1. Introduction

Nowadays, the majority of quantum key distribution systems (QKD) are implemented by utilizing weak coherent states. Examples are point-to-point [1,2] and twin-field systems with three parties [3,4,5,6]. Further examples are systems based on phase modulated multi-frequency states known as subcarrier wave QKD (SCW QKD) [7,8]. In addition, the state preparation approach utilized in SCW QKD can also be applied to a twin-field QKD system [9]. Different protocols can be implemented by using phase-modulated weak coherent states, including widely-used BB84-like protocol [10,11] (four phase states equally distributed in the phase plane with phase difference between neighboring state as $\pm \frac{\pi }{2}$), and the B92 protocol [12] (two phase states with phase difference ±π). The first SCW QKD setup has been proposed by the group of Merolla [13,14]; it implements B92 protocol with phase modulation. Thereafter, the implementation of BB84-like protocol has been demonstrated for the SCW scheme by the same group using amplitude modulation [15]. The replacement of phase modulation by amplitude modulation was necessary for decoding all states at the receiver’s side. In the last decade, the latter approach was expanded to the subcarrier multiplexing technique, which allows the authors of [16] to increase the key generation rate in several times. Another variation of the system was proposed in [17]. The authors show the possibility of implementing a strong reference technique by monitoring the intensity of the carrier wave. A version of the BB84-like protocol with phase modulation has recently been demonstrated by several authors of this work in [7,8,9,18]. Proposed in the latter papers approach allows to decode only one state in each <<basis>> (the word basis only means two states with the phase difference ±π). Moreover, recently it was shown that SCW QKD systems can utilize homo- or heterodyne detection schemes for continuous variable protocols [19].

Modulated multi-mode states are prepared by single frequency-mode coherent states, generated with laser beam, modulated by an electro-optical modulator. Detailed explanation of phase modulation’s quantum regime is given in [20], and the structure of phase modulated states is shown in Equation (1). Briefly, information coding is made by choice of the modulation signal phase (made by the sender) and decoding is based on the fact that detection event should only be in case of matching second modulation signal. The second modulation process is similar to interference. Our study describes the process of second modulation in the case of modulation indices mismatch resulting from difference in the orientation of signal polarization at the fist and the second modulations (in general effective indices of modulation depend on polarization); divergence of the orientation of signal polarization is introduced by propagation through optical fiber (OF). It also should be noted that since the construction of the experimental setups for discrete variable and continuous variable SCW QKD systems are quite similar, except for the detection method. Proposed analysis is suitable for both discrete and continuous types of variables (where the second modulation takes place).

The dielectric permittivity tensor in general is complex and anisotropic [21,22,23,24]. The real and the imaginary parts of the tensor determine birefringence and dichroism in the OF respectively; both phenomena also depend on the signal frequency. Changes in dielectric permittivity tensor of the OF may be caused by inhomogenities and impurities in the structure of the fiber or by physical impact on OF (bends and torsions) or by external impacts (temperature fluctuations, vibrations, magnetic fields, etc.). The above-mentioned phenomena affect the polarization state of the signal. Polarization mode dispersion and polarization dependent losses [25,26,27,28,29,30] in the OF are well studied, analysis can be made in linear quantum [31,32], linear classical [33,34,35], and nonlinear regimes [36,37,38]. It should be noted that quantum approach may reveal new features or even predict new quantum effects. However, the most important is its applicability in case of non-classical light (single photons, squeezed light, etc.). In this paper we use the approach that describes non-unitary dynamics of arbitrary quantum states developed in [39]. This model is applied to the case of multimode phase modulated weak coherent states in linear quantum regime; linearity requires the fact that states with low intensity are used.

Lindblad master-equation is the most general type of equations that can describe temporal evolution of an arbitrary quantum system that interacts with the environment. Considered master-equation can be applied to any class of problems. The special case of Lindblad master-equation in Markov-Born approximation [40] is the quantum Liouville equation; it it suitable for further consideration in the paper description of quantum non-unitary evolution of quantum states in OF channels. Physical properties of quantum states dynamics in OF can be easily connected with their information properties by convenient representation of the Liouville equation.

This paper is organized as follows. Expression of quantum bit error rate is derived in Section 2; effects of modulation indices mismatch is considered in the Section 2.1, polarization dynamics of quantum signal propagated through optical fiber is considered in Section 2.2, Section 2.3 combines results of previous ones. Numerical simulations and analysis of the dependencies are presented in Section 3. The discussion and recommendations presented in Section 4 conclude the paper.

## 2. Materials and Methods

### 2.1. Effects of Modulation Indices Mismatch

As it was stated earlier, this section will be based on the main approaches developed in [7,20]. It should be noted that we investigate an idealized model of phase modulation. In this model we disregard single photon detector dark counts, synchronization, and filtration systems imperfections. Thus, we simplify the model and hence we explicitly consider the influence of only optical fiber characteristics on the developed quantum bit error rate (QBER); it is obvious that additional imperfections introduce higher error rates.

Electro-optical phase modulator reallocates the energy between the interacting modes in such a way that a multimode state after the modulator can be expressed as

(1) $$|\psi \left(\phi_{A}\right)\rangle =\overset{S}{\underset{j=-S}{⨂}}{|\alpha_{j}\rangle }_{j}=\overset{S}{\underset{j=-S}{⨂}}{|\alpha_{0}d_{0j}^{S}\left(M\right)e^{-i\phi_{A}j}\rangle }_{j}$$

where $\phi_{A}$ is the phase of radio-frequency modulating signal, $\alpha_{j}$ is the amplitude of a coherent state at each mode after modulation, $\alpha_{0}$ is the initial amplitude of single-mode coherent state that enters modulator, $d_{0j}^{S}$ is the Wigner d-function commonly used throughout quantum angular momentum theory. Its argument is dependent on modulation index m as follows (approximately, taking into account large *S*):

(2) $$M=\frac{m}{S}$$

After the signal is transmitted through the fiber and then modulated for the second time the multimode state has the form:

(3) $$|\psi_{B}\left(\phi_{A},\phi_{B}\right)\rangle =\overset{S}{\underset{j=-S}{⨂}}{|\sqrt{\eta (L)}\alpha_{0}e^{-i\left(\theta_{0}+\theta_{1}j\right)}d_{0j}^{S}\left(M^{\prime }\right)\rangle }_{j}$$

where η(L) is optical system transmission coefficient dependent on the length of optical fiber, $\theta_{0}$ is phase obtained by transmission, $\theta_{1}$ is common phase, introduced by modulator (later on we disregard this phase). A new d-function argument is defined by the following relation considering the perfect case [41]:

(4) $$\cos\left(M^{\prime }\right)=\cos^{2}\left(M\right)-\sin^{2}\left(M\right)\cos\left(\phi_{A}-\phi_{B}\right)$$

If $\phi_{A}-\phi_{B}=0$ then the d-function argument is doubled $M^{\prime }=2M$. Meanwhile if $\phi_{A}-\phi_{B}=\pi$ then the d-function argument turns zero $M^{\prime }=0$. This case of $d_{0j}^{S}\left(0\right)=\delta_{0j}$ corresponds to total energy allocation back to central mode leaving vacuum states on the side bands. It looks like some kind of interference. In general the expression stated above can be rewritten as follows:

(5) $$\cos\left(M^{\prime }\right)=\cos\left(M\right)\cos\left(M\sigma \right)-\sin\left(M\right)\sin\left(M\sigma \right)\cos\left(\phi_{A}-\phi_{B}\right)$$

where σ corresponds to the mismatch coefficient of d-function arguments and hence of modulation indices (due to their proportionality).

As it can be seen from the above, the signal’s mean photon number that is incident on the single photon detector for the time ΔT can be obtained as a sum of mean photon number of each spectral component. We want to acknowledge that spectral filtration is needed in order to select the signal of interest on the side bands (central frequency does not contain any phase information). Thus, the optical power of signal is proportional to the following expression:

(6) $$n_{ph}\left(\phi_{A},\phi_{B}\right)=\sum_{j\neq 0}{\left|\sqrt{\eta (L)}\alpha_{0}e^{-i\theta_{0}j}d_{0j}^{S}\left(M^{\prime }\right)\right|}^{2}=\mu_{0}\eta \left(L\right)\left(1-{\left|d_{00}^{S}\left(M^{\prime }\right)\right|}^{2}\right)$$

And now let us investigate the expressions stated earlier with regard to several approximations. Firstly, standard fiber elecro-optical modulators are used for the SCW QKD; so the following approximations are valid in the limit S→∞ and (from Equation (2)) M→0. Consequently, the following approximations may be introduced:

(7) $$\begin{array}{c} \cos\left(M^{\prime }\right)\approx 1-\frac{{\left(M^{\prime }\right)}^{2}}{2}\\ \cos\left(M\right)\cos\left(M\sigma \right)\approx 1-\left(1+\sigma^{2}\right)\frac{{\left(M\right)}^{2}}{2}\\ \sin\left(M\right)\sin\left(M\sigma \right)\approx \sigma M^{2} \end{array}$$

Therefore, the Equation (4) is altered as:

(8) $${\left(M^{\prime }\right)}^{2}=M^{2}\left(1+2\sigma \cos\left(\phi_{A}-\phi_{B}\right)+\sigma^{2}\right)$$

Since $\frac{M}{m}=\frac{M^{\prime }}{m^{\prime }}$ (from Equation (2)) the above equation can also be written as follows:

(9) $${\left(m^{\prime }\right)}^{2}=m^{2}\left(1+2\sigma \cos\left(\phi_{A}-\phi_{B}\right)+\sigma^{2}\right)$$

Secondly, for the SCW QKD the approximation of a small modulation index, i.e., m<1 is valid. Then the mean photon number can be estimated as:

(10) $$\mu =\mu_{0}\eta \left(1-{\left|d_{00}^{S}\left(M^{\prime }\right)\right|}^{2}\right)\approx \mu_{0}\left(1-J_{0}^{2}\left(m\right)\right)\approx \frac{\mu_{0}m^{2}}{2}$$

As a consequence we express the optical power dependent on the difference between phases and amplitudes of modulation radio signals (defining mismatch of modulation indices) as follows:

(11) $$P_{\det}\left(\phi_{A},\phi_{B}\right)=\mu \eta \left(L\right)\left(1+2\sigma \cos\left(\phi_{A}-\phi_{B}\right)+\sigma^{2}\right)$$

### 2.2. Analysis of the Polarization Dynamics

We would like to investigate the Liouville equation as in [39] that describes non-unitary dynamics of quantum state ${\hat{\rho }}_{0}$ with regard to chosen polarization basis (H,V):

(12) $$\left\{\begin{array}{c} \frac{\partial \hat{\rho }\left(t\right)}{\partial t}=-i\left[\hat{H},\hat{\rho }\left(t\right)\right]+\hat{\Gamma }\hat{\rho }\left(t\right)\\ \hat{\rho }{\left(t\right)|}_{t=0}={\hat{\rho }}_{0} \end{array}\right.$$

where Hamiltonian is as follows:

(13) $$\hat{H}=\sum_{i\in H,V}\omega_{i}\left({\hat{a}}_{i}^{†}{\hat{a}}_{i}+\frac{1}{2}\right)$$

where $\omega_{i}$ is the optical signal frequency (with respect to basis), ${\hat{a}}_{i}$$\left({\hat{a}}_{i}^{†}\right)$ is the annihilation (creation) operator of a polarization mode. The relaxation superoperator that describes the thermalization process acting on the state’s density operator is as follows:

(14) $$\hat{\Gamma }\hat{\rho }=\sum_{i\in H,V}\frac{-\gamma_{i}}{2}\left(\left(n_{T}+1\right)\left({\hat{a}}_{i}^{†}{\hat{a}}_{i}\hat{\rho }+\hat{\rho }{\hat{a}}_{i}^{†}{\hat{a}}_{i}-2{\hat{a}}_{i}\hat{\rho }{\hat{a}}_{i}^{†}\right)+n_{T}\left({\hat{a}}_{i}{\hat{a}}_{i}^{†}\hat{\rho }+\hat{\rho }{\hat{a}}_{i}{\hat{a}}_{i}^{†}-2{\hat{a}}_{i}^{†}\hat{\rho }{\hat{a}}_{i}\right)\right)$$

where $\gamma_{i}$ is the thermalization rate (with respect to basis), $n_{T}$ is the mean number of thermal photons, which equals

(15) $$n_{T}=\frac{1}{e^{\frac{\hbar \omega }{kT}}-1}\;$$

where *ℏ* is reduced Planck’s constant, *k* is Boltzmann’s constant, *T* is environmental temperature (here we assume that both polarization modes have almost the same value of $n_{T}$ since it is small, e.g., $n_{T}\approx 10^{-13}$ for 1.55μ m and 300 K).

We introduce the Stokes parameters in order to estimate the degree of polarization of a weak coherent state (considering a single mode) after being transmitted through optical fiber. The Stokes parameters can be expressed as mean values of the following operators [42]:

(16) $$\begin{array}{c} {\hat{S}}_{0}={\hat{a}}_{H}^{†}{\hat{a}}_{H}+{\hat{a}}_{V}^{†}{\hat{a}}_{V}\\ {\hat{S}}_{1}={\hat{a}}_{H}^{†}{\hat{a}}_{H}-{\hat{a}}_{V}^{†}{\hat{a}}_{V}\\ {\hat{S}}_{2}={\hat{a}}_{H}^{†}{\hat{a}}_{V}+{\hat{a}}_{V}^{†}{\hat{a}}_{H}\\ {\hat{S}}_{3}=-i\left({\hat{a}}_{H}^{†}{\hat{a}}_{V}-{\hat{a}}_{V}^{†}{\hat{a}}_{H}\right) \end{array}$$

Then we may act by any of the stated above operators on the Equation (12) and apply the trace operation:

(17) $$\mathrm{Tr}\left(\hat{S}\frac{\partial \hat{\rho }\left(t\right)}{\partial t}\right)=-i\mathrm{Tr}\left(\hat{S}\left[\hat{H},\hat{\rho }\left(t\right)\right]\right)+\mathrm{Tr}\left(\hat{S}\hat{\Gamma }\hat{\rho }\left(t\right)\right)$$

Thus, after some algebraic actions, we obtain a system of equations describing the dynamics of the Stokes parameters (here we assume that the bandwidth of the signal spectrum is rather narrow, comparable to several GHz, and, as a consequence, optical fiber characteristics are almost equal to its different frequency components):

(18) $$\begin{array}{c} \frac{\partial S_{0}}{\partial t}=-\frac{\left(\gamma_{H}+\gamma_{V}\right)}{2}S_{0}-\frac{\left(\gamma_{H}-\gamma_{V}\right)}{2}S_{1}+n_{T}\left(\gamma_{H}+\gamma_{V}\right)\\ \frac{\partial S_{1}}{\partial t}=-\frac{\left(\gamma_{H}-\gamma_{V}\right)}{2}S_{0}-\frac{\left(\gamma_{H}+\gamma_{V}\right)}{2}S_{1}+n_{T}\left(\gamma_{H}-\gamma_{V}\right)\\ \frac{\partial S_{2}}{\partial t}=-\frac{\left(\gamma_{H}+\gamma_{V}\right)}{2}S_{2}-\frac{\left(\omega_{H}-\omega_{V}\right)}{2}S_{3}\\ \frac{\partial S_{3}}{\partial t}=\frac{\left(\omega_{H}-\omega_{V}\right)}{2}S_{2}-\frac{\left(\gamma_{H}+\gamma_{V}\right)}{2}S_{3} \end{array}$$

### 2.3. QBER Model

It should be noted that the choice of polarization basis (H,V) is determined in such a way that one of the basis vectors is parallel to the OF dichroism axis (i.e., making relaxation superoperator diagonal). However, modulators may be aligned in the other basis $\left(H^{\prime },V^{\prime }\right)$, we assume both of them are aligned by the $H^{\prime }$ direction. Then initial Jones vector of the state at the entrance to the first modulator is in the basis $\left(H^{\prime },V^{\prime }\right)$ and takes the form

(19) $$\left(\begin{array}{c} \alpha_{H^{\prime }}=1\\ \alpha_{V^{\prime }}=0 \end{array}\right)$$

We can establish the relation between two vectors:

(20) $$\left(\begin{array}{c} \alpha_{H}\\ \alpha_{V} \end{array}\right)=\left(\begin{array}{cc} \cos(\theta ) & -\sin(\theta )\\ \sin(\theta ) & \cos(\theta ) \end{array}\right)\left(\begin{array}{c} \alpha_{H^{\prime }}=1\\ \alpha_{V^{\prime }}=0 \end{array}\right)=\left(\begin{array}{c} \cos(\theta )\\ \sin(\theta ) \end{array}\right)$$

where θ is the angle between $\left(H^{{}^{\prime }},V^{{}^{\prime }}\right)$ and (H,V) bases.

The system of dynamic equations introduced in the previous section takes into account various physical phenomena. It is also necessary to determine the amount of optical power corresponding to each polarization mode. We want to acknowledge that the term $\frac{\left(\omega_{H}-\omega_{V}\right)}{2}$ stands for birefringence of OF. This parameter in general depends not only on physical characteristics of OF, but also on stress caused by curls, bends, torsions, etc. Thus, this parameter may vary significantly. In order to take this fact into account we assume that the system may contain a set of wave plates before the receiver’s block to balance out and hence disregard the birefringence. We can express assumptions stated above simply as $\frac{\left(\omega_{H}-\omega_{V}\right)}{2}=0$ and $S_{3}\left(0\right)=S_{3}\left(t\right)=0$. Hence, there is a two-dimensional solution in the $\left(S_{1},S_{2}\right)$ plain. Then optical power corresponding to each polarization mode can be defined as

(21) $$P_{H}=\frac{\left(S_{0}+S_{1}\right)}{2},\;P_{V}=\frac{\left(S_{0}-S_{1}\right)}{2}$$

We substitute the terms defined in (21) into the system (18) to find the solution:

(22) $$\begin{array}{c} P_{H}\left(t,\mu \right)=P_{H}\left(0,\mu \right)e^{-\gamma_{H}t}+\frac{n_{T}}{2}\left(1-e^{-\gamma_{H}t}\right)\\ P_{V}\left(t,\mu \right)=P_{V}\left(0,\mu \right)e^{-\gamma_{V}t}+\frac{n_{T}}{2}\left(1-e^{-\gamma_{V}t}\right) \end{array}$$

with

(23) $$P_{H}\left(0,\mu \right)=\mu {\left|\alpha_{H}\right|}^{2},\;P_{V}\left(0,\mu \right)=\mu {\left|\alpha_{V}\right|}^{2}$$

where μ is the mean photon number of the sidebands, $\alpha_{H}$ and $\alpha_{V}$ are Jones vector components in the corresponding basis. It should be taken into account that expressions in the Equation (22) have the form of power, not amplitude, while performing the reverse bases transform:

(24) $$\begin{array}{c} P_{H^{\prime }}\left(t,\mu \right)=P_{H}\left(t,\mu \right)\cos^{2}\left(\theta \right)+P_{V}\left(t,\mu \right)\sin^{2}\left(\theta \right)+\sqrt{P_{H}\left(t,\mu \right)P_{V}\left(t,\mu \right)}\sin\left(2\theta \right)\\ P_{V^{\prime }}\left(t,\mu \right)=P_{V}\left(t,\mu \right)\cos^{2}\left(\theta \right)+P_{H}\left(t,\mu \right)\sin^{2}\left(\theta \right)-\sqrt{P_{H}\left(t,\mu \right)P_{V}\left(t,\mu \right)}\sin\left(2\theta \right) \end{array}$$

Here we define the following domain $0\leq \theta \leq \frac{\pi }{2}$. Initial state is aligned by $H^{\prime }$ then it is modulated by $m_{H^{\prime }}$. However, due to propagation there might be non-zero $P_{V^{\prime }}\left(t,\mu \right)$ component that should be modulated second time with different modulation index $m_{V^{\prime }}$ (that depends on signal polarization). Thus we introduce $\sigma =\frac{m_{V^{\prime }}}{m_{H^{\prime }}}$. The second modulation leads to the increase or reduction in mean photon number depending on modulation signal phase difference as well as on the modulation indices difference, according to the Equation (11). There are four possible cases (it is implied that the equipment possesses no imperfections, in order for us to estimate the contribution from polarization divergence only):

1. Polarization of signal coincides with orientation of effective modulation, phase difference equals zero: μ→4μ,
2. Polarization of signal coincides with orientation of effective modulation, phase difference equals π: μ→0,
3. Polarization of signal does not coincide with orientation of effective modulation, phase difference equals zero: $\mu \to \mu {\left(1+\sigma \right)}^{2}$,
4. Polarization of signal does not coincide with orientation of effective modulation, phase difference equals π: $\mu \to \mu {\left(1-\sigma \right)}^{2}$.

It is important to comment on the modulation of thermal light that appears in the fiber. We assume that there is no thermal light at the first modulation (t≈0). Thus, the second modulation only reallocates the energy of the thermal photons as if it is only the first modulation. In other words, the second modulation does not affect the mean value of thermal light photons in the spectrum.

Taking into account all the previous reasoning, one may derive a final expression for QBER (that is the ratio of undesirable detection events when the phase difference equals π to the total amount of detection events; detection events rates are proportional to optical power of the signal in Mandel linear approximation [21], that can be applied taking into account μ<1 and additioanl losses):

(25) $$Q\left(t\right)=\frac{P_{H^{\prime }}\left(t,0\right)+P_{V^{\prime }}\left(t,\mu {\left(1-\sigma \right)}^{2}\right)}{P_{H^{\prime }}\left(t,0\right)+P_{V^{\prime }}\left(t,\mu {\left(1-\sigma \right)}^{2}\right)+P_{H^{\prime }}\left(t,4\mu \right)+P_{V^{\prime }}\left(t,\mu {\left(1+\sigma \right)}^{2}\right)}$$

We also want to explicitly show the relation between time-dependent losses and OF-length-dependent losses:

(26) $$e^{-\frac{\left(\gamma_{H}+\gamma_{V}\right)t}{2}}=10^{\frac{\alpha L}{10}}$$

where α is specific loss coefficient (dB/km), $L=\frac{ct}{\beta }$ is the distance that optical signal passes in the OF, *c* is the speed of light, β is propagation constant. We want to define relative loss difference for two polarization modes for the purpose of accurate OF dichroism description:

(27) $$\Delta =\frac{\gamma_{H}-\gamma_{V}}{\gamma_{H}}$$

Then we get:

(28) $$\begin{array}{c} \gamma_{H}=\frac{-2\alpha c}{\beta \left(2-\Delta \right)10\ln\left(10\right)}\\ \gamma_{V}=\gamma_{H}\left(1-\Delta \right) \end{array}$$

## 3. Results

We want to examine Equation (25) for the typical values of the single mode OF characteristics. We choose the standard telecommunication OF suitable for transmission of a signal with wavelength 1550 nm (like SMF-28). Then α=−0.2 dB/km, $c=3\cdot 10^{5}$ km/s, β=1.5. We do not assume Δ as it will be considered as a further parameter (this parameter can be determined with standard polarimetric measurements). According to Equation (28) for these parameters we get $\gamma_{H}=1829$ Hz. For temperature of 300 K and wavelength 1550 nm we estimate $n_{T}=10^{-13}$. Parameter θ can be determined with standard polarimetric measurements based on the system’s construction. Thus, it is not a typical parameter and its value may be arbitrary. Parameter σ is determined with standard polarimetric measurements as well for a particular electro-optical modulator or is defined in a technical passport. We acknowledge that the value of this parameter may vary significantly as it is dependent on the type of nonlinear crystal used in particular modulator and modulator design in general. As a result, for some modulators the value of the modulation indices ratio may be close to one, for others close to zero.

Equation (25) seems to be a bit complicated for direct analysis. Thus, we would like to introduce approximated expression (second order of *t*) for Equation (25) as follows:

(29) $$Q_{app}\left(t\right)=\frac{t^{2}}{64\mu^{2}{\left(1-\sigma \right)}^{2}}\left(\gamma_{H}\left(1-\Delta \right)n_{T}\cot\left(\theta \right)+\mu \gamma_{H}\Delta {\left(1-\sigma \right)}^{2}\sin\left(2\theta \right)+\gamma_{H}n_{T}\tan\left(\theta \right)\right)$$

**Figure 1:**
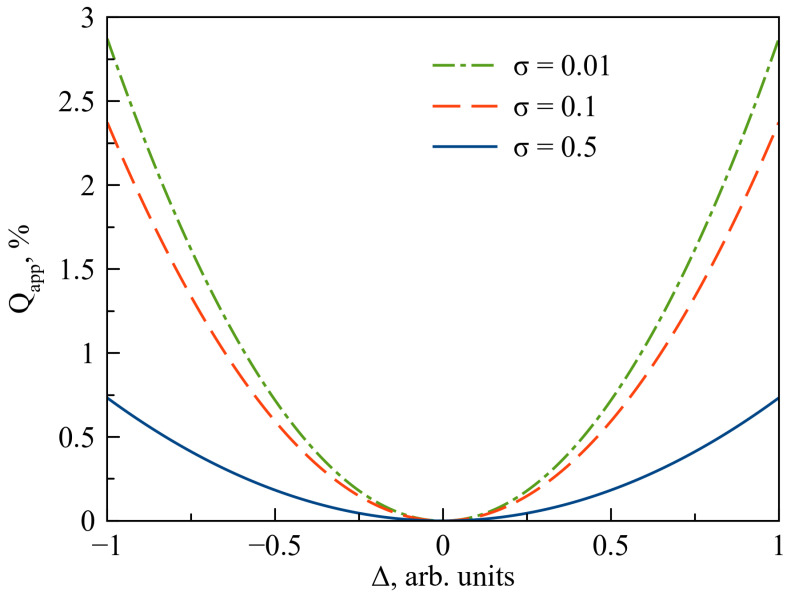
Dependence of approximate maximal value of quantum bit error rate (QBER) on Δ that characterized dichroism at 150 km OF length for several values of σ. For even smaller values of σ dependence does not sufficiently increase QBER contribution. Typical values of Δ are about ±0.1 so we may conclude that the considered effect is weak.

This approximation works well (relative divergence is less than few percents) up to around 150 km (recalculating propagation time into length of the OF by $L=\frac{ct}{\beta }$). We estimate the maximal contribution to the QBER at 150 km by choosing appropriate θ that maximizes the value for different Δ. The obtained dependence is shown in Figure 1. Contribution of dichroism was found to be weak; even for extreme values of Δ it is no more than 3%. Typical values of Δ are about ±0.1 then the maximal expected contribution will be no greater than tenth percents. Despite that in case of fine tuning of the system (e.g., 1–1.5% QBER) even the small contribution is worth being concerned. Obviously in general the contribution of imperfect devices will be more tangible. Imperfect spectral filtration provides constant contribution to the QBER and detector’s dark count become crucial at high distances. Typical tolerable QBER for SCW QKD systems is about 5–7%. The upper bound for QBER is in general defined by hardware and software characteristics, hence it may vary.

## 4. Discussion

Based on the quantum theory of electro-optical modulator and the quantim theory of open systems we developed a model that describes the influence of polarization divergence of a signal propagating through an OF on QBER for SCW QKD.

It was shown that the polarization divergence can be viewed as two separate effects. The first one is due to birefringence and it can be compensated for by waveplates. However, there is also a second dichroism-related effect that cannot be compensated for in any way. It was explicitly demonstrated that QBER is dependent on Δ that indicates dichroism. Fortunately, analysis reveals that dichroism-related contribution for QBER is rather weak. It should be stated that the development of this model was motivated mostly by the necessity of minimizing QBER for the purpose of the increase of the efficiency of SCW QKD protocol performance. Even a few percent of the considered contribution to QBER is worthy of concern.

The simplest way of minimizing QBER resulting from polarization divergence is the utilization of electro-optical modulators whose modulation efficiency is not dependent on polarization of the optical signal. In addition, there is still birefringence that should be taken into account. Thus, it is necessary to compensate for this negative effect. It can be performed with the help of active [43] or passive [8] additional subsystems of compensation of polarization distortion. However, the additional optical parts of the scheme provide higher losses at the receivers (Bob) block and hence decrease the performance of the system.

All things considered, the above work may be summarized by the fairly simple conclusion that utilization of polarization-independent schemes is necessary for the maintenance of sustainable SCW QKD system operation. Based on the results of our study, the authors may recommend utilizing electro-optical phase modulators whose modulation efficiency is not dependent on polarization of the modulated optical signal or additional subsystems of compensation of polarization distortion; however, the former is slightly preferable.

## Abbreviations

The following abbreviations are used in this manuscript:

QKD	Quantum Key Distribution
SCW	Subcarrier Wave
OF	Optical Fiber
QBER	Quantum Bit Error Rate
